# Remarks by Prof. Huxley to the London Dental Students

**Published:** 1877-09

**Authors:** 


### ARTICLE III.

### *Remarks by Prof. Huxley,*

**At the Distribution of Prizes to the Students of the London School of Dental Surgery.**

We print these remarks of Prof. Huxley because they are characterized by that rare good sense which we would *a priori* expect from one who enjoys so enviable and world-wide a reputation as a scientist, a scholar and an educator, and also because these views are directly at variance with views which sometimes have been published in dental journals of this country, but which, I am happy to be able to state, are held but by very few of those whose opinions are worthy of any particular attention. We are admonished also by these remarks of Prof. Huxley's that the dental profession of Great Britain is making rapid strides in establishing dental colleges and providing educational facilities for students; and from present appearances we must conclude that our boasted superiority in dentistry will not long be retained:

LADIES AND GENTLEMEN: The ordinary duties of a person in my position have been lightened to-day by a practice, which in the interests of chairmen in general I should like to see made universal, of committing the statement of the merits of the various gentlemen who have distinguished themselves in their studies to their teachers and to other persons who are eminent in the departments of knowledge in which they have won so much credit. They have had that which is said to be the highest form of praise—the praise of men who are themselves worthy of praise; and we have all wished them that success in their future career which is inevitable, if they pursue their calling in life with the same diligence and energy and single-minded sincerity of purpose as has characterized their student life. There remains, therefore, one would almost think, nothing for me to do; and I wish that I could take that view of my functions, because then I should, for once in my life, feel perfectly confident that I should discharge my duties successfully. But I observe that on the programme of arrangements it is said that I shall give an "address"—far too formal a title, let me say, for the few words which I shall have the honor of placing before you: and I must undertake that task with as much brevity as is consistent with saying what I wish to say. The speakers who have gone before me have very justly and rightly enlarged upon the great importance to all of us of that branch of the medical profession which is taught in the London School of Dental Surgery. In fact, the teeth reminds us of their existence throughout the whole seven ages of man's life; the "mewling and puking in the nurse's arms" being not unfrequently referable to coming teeth, and the precipitation of that last stage—of the finale of it at least—which Shakespeare characterizes as "sans eyes, sans teeth, sans everything," being often directly aided by their absence. It would be hard to say in our present state of civilization, and apparently even in times before civilization, how large a share in the welfare and misery of mankind has been played by these organs, which remind one of some kinds of friends who are always plaguing us while they live and whose real merits and value only become perfectly clear when they have departed. Hence it is that I can understand why so large a proportion of the general public should have made its appearance at the meeting to-day, for undoubtedly there are few professions in which the general public has so direct and so immediate an interest as in that accomplishment which is promoted by such institutions as those to which the students whose merits you have seen recognized to-day belong.

I made it my business to-day to pay a hasty visit—I am sorry to say too hasty a one—to the London School of Dental Surgery. I saw busily carried out those operations of which most of us think with a certain shudder, unless we recollect perhaps that it is almost worth the pain of having a tooth out, to know the extreme bliss of having got rid of the toothache. (Laughter.) I visited what corresponds with the wards in other hospitals and saw the array of those wonderful chairs which takes the place of beds in ordinary hospitals; I examined the excellent lecture room and the museum in which the student can acquaint himself with the forms of healthy and of diseased teeth. I observed there with some surprise, for the moment, skulls of hippopotamuses and skulls of rhinoceroses which I thought were rather more in my way than in that of the Dental Hospital, and I had for an instant a kind of vague feeling that the skill of the modern Dentist had now extended so far that, like the ancient Alexander, he sighed for new worlds to conquer, and leaving the grinders of man had betaken himself to the tusks of the hippopotamus and the molars of the elephant. But I learnt subsequently that these preparations were for the illustration of that valuable part of the instruction in the Dental College which my friend Professor Flower alluded to just now as being given by Mr. Tomes. And there was one very curious specimen in the museum which I will mention to you, as indicating in a rough way the enormous progress which has been made during the last century in medical science and in medical art, as in all other arts and sciences. In an obscure corner of the museum—too obscure, in fact—I saw a piece of wood having upon it this inscription: "Thomas Smith lets blood, draws teeth, handles teakittles." That led one back, ladies and gentlemen, to a state of the profession of Dentists which corresponded, in comparison with its present state of complication, with the time of the men with stone implements as compared with those vast armaments with which two great nations are now preparing to decimate one another; and the enormous interval between the rude apparatus by which Thomas Smith performed his operations and the exquisitely skillful appliances by which the modern operations of Dentistry are performed, will give you some impression of the amount of study and of knowledge which is required at the hands of the modern practitioner of Dentistry—of that amount of study and of knowledge the means of pursuing and acquiring of which are so amply and so well given in the School of Dental Surgery of London. It has been remarked by a previous speaker that the art of the Dentist, although confined to one particular set of organs in the human body, must necessarily extend over the rest of the bodily frame, inasmuch as those troublesome organs, the teeth, being part of the living organism, and themselves alive, are inseparably connected with all other parts of the body, and act upon them and react upon them; so that no man can hope to be an intelligent or, indeed, a competent practitioner of the Dental branch of the medical profession who has not a good and sufficient grounding in general medicine and in general surgery. Without that he must needs be an empiric in the worst sense, inasmuch as he will be meddling with a complicated machine the work of which he does not in the slightest degree comprehend. But the slightest attention to modern Dentistry must show that this branch of the medical profession is, in a very strict sense, a specialty. I do not merely mean to say that it is a specialty in the sense in which the treatment of this, that, or the other internal organ may be a specialty, but it is a specialty in an even more perfect sense than the treatment of the diseases of the eye is a specialty. I mean that for its successful practice it requires an amount of technical knowledge and of skill in the use of appliances which can only be gained by a special and peculiar training, so that those who look, as the founders of the School of Dental Surgery have done, at that liberalization of the profession which consists in making a man thoroughly know what he is about, have very rightly discriminated the two elements in the education which is to be given, one of them what we may call the general medical element, the other element special to this particular branch of the profession. The means of obtaining general medical education are abundant and are to be met with in the numerous medical schools which are scattered over London; and, therefore, very wisely, as I venture to think, the founders of this school did not propose to include into that instruction the general medical and surgical teaching which could be better got elsewhere in connection with the great hospitals. And they refer their students to these great hospitals for such instruction as is required, But, on the other hand they have considered—and here, again, I cannot but think wisely—that it would be quite impossible, or at least that it would be very hard for hospitals devoted to general purposes to be expected to possess the many special appliances which are needed for instruction in the technical part of the Dentist's craft. And therefore they tell their students to acquire their general knowledge where it is best to be acquired, and then to come to this special school of Dental Surgery for the purpose of obtaining that special and peculiar kind of knowledge which is not to be had elsewhere. It does not appear to me that that seems a very sensible and wise recognition of the principle of the division of labor. I would beg you to remark that in doing this the founders of this school have not had the mistaken idea of separating the profession of the Dental Surgeon from other branches of the medical profession. They require of their students, in the first place, the common elements of the education of an enlightened man. The preliminary examinations, as I understand, are common to their curriculum and that of the Royal College of Surgeons. In the second place, as I have said, they require a knowledge of general medicine and general surgery—without wasting the time of their students by carrying them into branches of study which can have no direct bearing upon their future career. And then, finally—and herein I think the wisdom of their arrangements is particularly shown—they do insist that the man who calls himself a Dental Surgeon—who has the diploma and the qualification for the practice of Dental Surgery, shall show by the length of time under which he has undergone instruction, by the courses through which he has passed, and finally by the results of examination, that he really does know that particular business. It so happens, ladies and gentlemen, that I have had at various times to express opinions respecting professional education. It has been my lot to serve on various commissions, university and other and to be obliged to acquaint myself somewhat carefully with what is doing in these matters, and I have heard a great deal of talk about what is called a liberal education. There is no man who has greater respect for a liberal education, in the proper sense of the word, than I have. There is no one who can have less inclination to despise any form of knowledge whatever. But another consideration has been very strongly forced upon me; that is, that in the conditions of life in this country, in the midst of the constant driving and pressure which is put upon all men and especially now-a-days upon young men, the persons who are entering a profession can only give a certain limited time to their pursuits, and that if they are to be made competent, if they are really to know what they are supposed to know, you must limit that which you require from them, and you must concentrate their energies upon those studies which it is absolutely needful for them to have well in hand. If not you may get very accomplished men —very versatile persons, but they will not have that confidence of their patients which is the foundation of all sound living in any profession whatever. They may be very liberally educated persons, but they will not be good practitioners, and I do hold that the first clear moral duty which every man who sets himself up before the public as able to do a certain thing has laid upon him is, that he shall be able to do that thing whether he can do anything else or not. Now, it is exactly upon that ground that I venture to think that the London School of Dental Surgery commends itself to our best wishes and to our warmest sympathies. By its regulations, the school does its best to secure that the members of the profession shall be cultivated persons, with the sort of sympathies with their patients and with their fellow-creatures in general which are alone to be gained by some elements of liberal culture. In the next place, it secures their having that general knowledge of medicine and surgery, to which I have referred as being absolutely essential to the intelligent practice of their profession ; and, in the third place, by that thorough practical training—that complete discipline in the actual work of their calling—which the students have the great advantage of obtaining in the hospital, they do their best to secure that the practitioner, when he leaves them shall be, in the best sense of the word, a liberally educated man, that is to say, a man who can honestly do the thing which he undertakes to do. I do not think, ladies and gentlemen, that I need detain you any longer. We have wished all prosperity to those gentlemen whose merits have been placed before us, and I think we should conclude with a hearty expression of good will towards, and good wishes for the prosperity of, an institution which is capable of yielding such admirable results.—*British Journal of Dental Science.*

